# Immunosenescence and Its Hallmarks: How to Oppose Aging Strategically? A Review of Potential Options for Therapeutic Intervention

**DOI:** 10.3389/fimmu.2019.02247

**Published:** 2019-09-25

**Authors:** Anna Aiello, Farzin Farzaneh, Giuseppina Candore, Calogero Caruso, Sergio Davinelli, Caterina Maria Gambino, Mattia Emanuela Ligotti, Nahid Zareian, Giulia Accardi

**Affiliations:** ^1^Laboratory of Immunopathology and Immunosenescence, Department of Biomedicine, Neuroscience and Advanced Diagnostics, University of Palermo, Palermo, Italy; ^2^Molecular Medicine Group, Department of Hematological Medicine, School of Cancer & Pharmaceutical Sciences, The Rayne Institute, King's College London, London, United Kingdom; ^3^Department of Medicine and Health Sciences “V. Tiberio”, University of Molise, Campobasso, Italy; ^4^Department of Epidemiology, Harvard T.H. Chan School of Public Health, Boston, MA, United States

**Keywords:** aging, immunosenescence, immunomodulation, immunotherapy, nutrition

## Abstract

Aging is accompanied by remodeling of the immune system. With time, this leads to a decline in immune efficacy, resulting in increased vulnerability to infectious diseases, diminished responses to vaccination, and a susceptibility to age-related inflammatory diseases. An age-associated immune alteration, extensively reported in previous studies, is the reduction in the number of peripheral blood naïve cells, with a relative increase in the frequency of memory cells. These two alterations, together with inflamm-aging, are considered the hallmarks of immunosenescence. Because aging is a plastic process, it is influenced by both nutritional and pharmacological interventions. Therefore, the role of nutrition and of immunomodulation in immunosenescence is discussed, due to the multifactorial influence on these hallmarks. The close connection between nutrition, intake of bioactive nutrients and supplements, immune function, and inflammation demonstrate the key role of dietary strategies as regulators of immune response and inflammatory status, hence as possible modulators of the rate of immunosenescence. In addition, potential options for therapeutic intervention are clarified. In particular, the use of interleukin-7 as growth factor for naïve T cells, the function of checkpoint inhibitors in improving T cell responses during aging and, the potential of drugs that inhibit mitogen-activated protein kinases and their interaction with nutrient signaling pathways are discussed. Finally, it is suggested that the inclusion of appropriate combinations of toll-like receptor agonists may enhance the efficacy of vaccination in older adults.

## Introduction

People worldwide are living longer. In 2025, there will be about 1.2 billion people over the age of 60, increasing to 2 billion by 2050 (1). However, the increase in lifespan does not coincide with the increase in healthspan, i.e., the period of life free from serious chronic diseases and disability. In fact, the influence of aging on humans is responsible for physiological dysfunctions in the different tissues, organs, and systems, including the immune system (2, 3). The age-related involvement of immune system leads to a progressive reduction in the ability to trigger effective antibody and cellular responses against infections and vaccinations. This phenomenon, called immunosenescence, a term coined by Roy Walford, is multifactorial, and affects both natural and acquired immunity, although T lymphocytes are dramatically affected (4). In fact, aging process more extensively affects acquired immunity than innate immunity (3, 5). Several factors, such as genetics, nutrition, exercise, previous exposure to microorganisms, biological and cultural sex, and human cytomegalovirus (HCMV) status can influence immunosenescence (3, 6–11).

Concerning sex, steroid hormones, linking to specific receptors, differentially modulate the immune system. In general, while estrogens increase the immune response, progesterone and androgens have immune suppressive actions. However, a few studies have analyzed the post-menopausal immune system (12). Therefore, it is unclear whether age-related changes in the immune system are different between men and women, although some data show that immunosenescence develops earlier in men than in women. This has been related to longer life expectancy of women (8, 13, 14). In addition, no evidence exists that males and females respond differently to therapeutic intervention against immunosenescence.

Many studies have emphasized the importance of viruses, such as herpes viruses, responsible for both latent and chronic infections, in shaping T cell compartments during aging (15). In particular, HCMV seropositivity seems related to many functional T cell changes. HCMV status has a greater impact than age on the immune system, because the virus contributes to shape the immune profile and function during normal human aging (16–18).

Understanding mechanisms of age-related disorders in immune regulation is important to identify more efficient strategies for immune rejuvenation and for effective induction of vaccination-mediated immunity in older individuals. Aging is a malleable process, affected by both nutritional and pharmacological interventions (19, 20). Therefore, immune system might also be prone to intervention. However, all possible therapies aimed at non-specifically “rejuvenating” the immune system might be counterproductive. In fact, the different parameters observed between young and older people could also be a product of the adaption vs. the exposome, i.e., all the stimuli that the immune system has undergone during life. Therefore, a targeted intervention for safe “rejuvenation” of the immune status in older people should be necessary (21).

Within the past years, numerous studies of underlying mechanisms of age-related immune decline have laid the groundwork for the identification of targeted approaches (5, 22–24). We will discuss below the most relevant strategies currently being investigated. We will also considered the role of nutrition in immunosenescence and in its counteraction in the section on dietary strategies currently being investigated. Further, we will examine the available data on growth factors [i.e., on interleukin (IL)-7], on monoclonal antibodies (MoAbs) that affect immune checkpoints, and on drugs that inhibit mitogen-activated protein kinases (MAPK) and their interaction with nutrient signaling pathways. These treatments, representing a promising therapeutic approach, will be treated in the section on clinical approaches. In the section on the other approaches in development, we will suggest that the inclusion of appropriate combinations of toll-like receptor (TLRs) agonists might enhance the efficacy of vaccination-mediated immunity in older adults. Finally, at the end of conclusion, we will outline possible future approaches.

## Summary of Immunosenescence

### Innate Immunity

The general picture of innate immunity in older people, which emerges from several studies, is that of the down-regulation of some functions and the up-regulation of others. We will discuss data on dendritic cells (DCs) due to their relevance for the immunotherapeutic approaches, including vaccination. For the other aspects of innate immunity in older individuals, see (3, 25, 26). Briefly, natural killer (NK) cell cytotoxicity is well-preserved in centenarians, and an increase in the actual number of NK cells is observed in healthy aging. Neutrophils show reduced function in bacteria phagocytosis and in the oxidative burst while macrophages show reduced chemotaxis and phagocytosis, and decreased cytokine production.

DCs, the most potent antigen presenting cells (APCs), can be divided into three subsets according to the expression of various markers (CD123, CD1c, CD141), one subset of plasmacytoid DCs (pDCs) and two subsets of myeloid DCs (mDCs) (27). Both pDCs and mDCs express TLRs that recognize conserved pathogen-associated molecular patterns (PAMPs) on microbes, and are key regulators of antimicrobial host defense responses. The type of TLR-activated DC determines the cytokine pattern (28).

There are discordant data on age-related changes in the frequency and absolute number of pDCs and mDCs. Regarding the ability to secrete cytokines upon stimulation, there are apparent inconsistencies in the available data for mDCs from older population. pDCs are instead characterized by a marked impairment of cytokine release in older people (27, 29). Recognition of microbial components by TLRs culminates in the secretion of type I interferons (IFNs) and cytokines that facilitate the coordination of innate to acquired immune responses. Peripheral blood mononuclear cells (PBMCs) isolated from older individuals (≥65 years) exhibited a delayed and altered response to stimulation with TLR agonists compared with cells obtained from young adults (≤ 40 years). This delayed response to agonists results in the reduced production of cytokines and chemokines (29). On the other hand, the addition of PAMPs to a subunit vaccine, triggering their corresponding pattern-recognition receptors (e.g., TLRs) improves vaccine efficacy in older humans and mice (25, 30–32). Accordingly, DCs together with naïve T cells represent the most restrictive elements for the immune response to primary viral infections in older people (33).

As the expression of TLRs remains constant during life, defects in signal transduction should be responsible for this impairment, as discussed by (24).

### Acquired Immunity and the Hallmarks of Immunosenescence

The quality and quantity of the T and B cell responses change with increasing age, with consequent changes on the effectiveness of the immune response. This leads to an inadequate immune response against newly encountered antigens. The apparently inevitable consequence of this complex scenario is the reduced ability of older individuals to respond to novel antigens and to vaccines, resulting in an increased susceptibility to infection and in the development of age-related diseases, including cancer (3). As critically reviewed by (3, 6, 16), a number of longitudinal studies of octogenarians and non-agenarians performed in Sweden defined an immunological risk phenotype (IRP). Participants with the reversal of CD4/CD 8 T-cell ratio, a reduced proliferative response to mitogenic stimuli, and severe reduced B cells number showed reduced survival. Subsequently, the data were implemented and related to HCMV seropositivity, because HCMV seropositivity is closely related to the reversal of CD4/CD 8 T-cell ratio. In fact, as discussed below, persistent HCMV infection leads to chronic stimulation of CD8 T cells, which expand clonally showing an effector memory phenotype characterized by low CD28 expression. The IRP was present in around 15% of 85-years-olds in these studies at baseline. Follow-up of 2-, 4-, and 6-years mortality revealed significantly higher all-cause mortality in the IRP group than in the majority of other octogenarians and non-agenarians. However, this IRP was not confirmed in the Leiden 85-Plus study, a prospective population-based cohort study of individuals at the age of 85 years living in Leiden (NL). Thus, immune parameters associated with survival may vary in diverse populations at different ages (6). Therefore, we focus on the changes we have considered the hallmarks of immunosenescence, based on the literature data (6, 23).

The hallmarks of immunosenescence include: (i) a reduced ability to respond to new antigens; (ii) the accumulation of memory T cells; (iii) a lingering level of low-grade inflammation termed “inflamm-aging.” Mechanistically, immunosenescence is only partially explained by organismal and cellular senescence. Therefore, these hallmarks of immunosenescence would be markedly affected by the history of the individual exposure to pathogens (6, 23).

The reduced ability to respond to new antigens is linked to a decreased number of peripheral naïve T and B cells (see last paragraph of this section). Naïve T cells are abundant in youth but may become “used up” by exposures to microorganisms over the course of life, hence differentiating into memory lymphocyte subsets. In addition, their number decreases following the involution of primary lymphoid organs, because age-related defects have been observed in their stroma. Some changes occur early in the developmental progression from hematopoietic stem cells (3). Thymus involution occurs at the time of puberty, and is characterized by atrophy and replacement by adipose tissue. This process seems related to the increase of sex hormones and to the decrease of IL-7, a hematopoietic growth factor secreted by stromal cells in the bone marrow and thymus. IL-7 exerts its action through the binding to a heterodimeric receptor composed of an α chain (IL-7Rα or CD127) and the common cytokine receptor γ chain (γc or CD132). CD127 is expressed on lymphoid lineage cells at different stages of development, whereas CD132 is shared with other cytokine receptors and expressed on most hematopoietic cells (34, 35). Irrespective of thymic activity, the naïve compartment only moderately decreases in size during the following life decades, while mostly maintaining overall diversity and distribution of clonal sizes. An abrupt contraction is seen in later life. Therefore, at the age of 50, T cell production is <10% of its previous peak levels. From an evolutionary point of view, this occurs because exposure to new pathogens is maximal during the first years of life, but less likely in later life when immune memory for previously encountered pathogens is both more prevalent and more significantly important for survival (3, 22, 36).

The life-long chronic antigen load causes the filling of the immunological space by a population of T lymphocytes with a late-differentiated phenotype and the shrinkage of the T cell repertoire. As previously stated, an age-related decrease in absolute number of peripheral blood naïve T cells is consistently found in all studies and in different human populations (22, 37). Due to the lifelong and chronic exposure to pathogens, T cells replicate several times and become late-differentiated effector memory T cells with features of replicative senescence (38). T cell senescence focuses on the phenotypic characteristics of individual lymphocytes and refers mainly to a low proliferative activity (39). Aging *per se* leads only to a relative accumulation of memory cell subsets, linked to the decrease in naïve cell populations. The absolute increase in memory T cells, called memory inflation, is observed only in older people infected by HCMV (40). These T cells do not express the co-stimulatory molecule CD28, required for the activation of T cells. The loss of CD28 occurs following cell proliferation, according to the observation that the CD28^−^ T cells have shorter telomeres than CD28^+^ cells. These CD28− cells express high levels of the adhesion molecule integrin CD11a/CD18 and have high levels of perforin and granzyme, responsible for the killing of the target cells. CD28 seems a good biomarker of immunosenescence, as further suggested by findings that late-differentiated CD8^+^/CD28^−^ T cells tend to accumulate particularly in older people, frail or affected by age-related diseases. These cells display a highly differentiated phenotype, expressing CD27, another co-stimulatory molecule, but not CD28 (however, in CD28^+^ subset, CD28^−^CD27^−^ seem to be more frequent). They also carry short telomeres, lack telomerase and express negative signaling receptors, such as programmed cell death protein (PD)-1, which is involved in the down-regulation of the immune system (see paragraph on checkpoints inhibitors; the example of PD-1 and CTLA-4). Senescent T cells also express CD57 displaying a high cytotoxic potential, and killer cell lectin-like receptor subfamily G member 1. Late-stage memory senescent T-cells may also acquire new functions, such as suppressive activity, as demonstrated *in vitro*. In addition, they are producers of pro-inflammatory cytokines (17, 18, 41–47). However, a longitudinal study of 249 research participants followed for 10 years has strongly suggested that HCMV infection is not a primary causative factor in the age-related increase in systemic inflammation (48). Therefore, the accumulation of memory T cells, especially late-stage differentiated CD8^+^ cells is viewed as the result of depletion of the reservoir of naïve cells over time by contact with pathogens and their conversion to memory cells. However, the memory responses can be unsustained, because T cell memory established in humans during early age can deteriorate during the second half of life. The most obvious example of unsustained memory responses is the reactivation of latent varicella zoster virus (VZV) infection that manifests as herpes zoster. A steady decline of VZV-specific CD4^+^ T cells over time has been documented, which is only very transiently boosted with zoster vaccination or reactivation (49). In contrast, high frequencies of antigen-specific T cells reactive to HCMV persist throughout life. T cell clones specific for HCMV dominate the repertoire in the older people and contribute to the contraction in diversity in the memory compartment (23).

Nearly 20 years ago, Looney et al. reported the dramatic impact of HCMV on the immune system of older people (50). This observation was subsequently described in numerous other studies (18, 46). In the latent state, the intermittent production of viral antigens prevents contraction of virus-specific T cells. Therefore, the virus is responsible for the generation of a large population of HCMV-specific CD8^+^ T cells, with a significant increase in highly differentiated CD8^+^ effector memory T cells, which expand clonally showing an effector memory phenotype characterized by low CD28 expression. As previously stated, this determines the phenomenon of memory cell inflation, leading to the emergence of vast populations of resting effector CD8^+^ and, to a lesser extent, CD4^+^ cells. In older people, one or a few clonal populations can occupy more than 25% of the entire CD8^+^ cell pool (46, 51). These inflated HCMV-specific memory T cells maintain their efficient effector functions for the lifetime of the individual (40, 46, 52). Inflationary CD8^+^ cells, after proper activation stimuli, can divide, secrete cytokines, and execute cytolysis, i.e., they are not exhausted. However, there may be a slight loss of control of HCMV replication in older compared with younger people. In fact, HCMV load in blood markedly increases in healthy people over the age of 70 years (53). Immune changes associated with HCMV may have significant impact during co-infection and vaccination, as well as on general and immunological fitness. However, the correlation between HCMV positivity and impaired responses is controversial because this relationship is observed in some but not all studies (54–56).

Persistent antigenic challenges lead to a poor response to newly encountered microbial antigens, as well as to a shift in the immune system toward an inflammatory, autoimmune, T helper (Th) 2 profile. In addition, the long-term chronic microbial burden induces progressive activation of macrophages, hence contributing to the chronic state of low-grade inflammation, inflamm-aging, another hallmark of immunosenescence (3, 9, 57). This term defines the systemic state of chronic low-grade inflammation considered a central biological pillar of the aging process and a common pathogenetic mechanism of age-related diseases, as well as a worse prognostic factor for all causes of death (9, 57–59). In the course of aging, there is a reduction in the ability to endure consequences of antigenic, chemical, physical, and nutritional triggers of inflammation. Chronic and low-grade inflammation can lead to tissue dysfunction and degeneration. Our immune system is quite efficient in fighting acute infections in young people, but not particularly efficient in responding to chronic stimuli, especially when they occur late in life. This leads to an increased production of pro-inflammatory cytokines and acute phase proteins (59, 60). Oxidative stress also plays an important role in determining and maintaining this low-grade inflammation, which, in turn contributes to oxidative stress (61, 62). Inflamm-aging results from the activation of signaling networks critical to inflammation, such as those regulated by the nuclear factor kappa-light-chain-enhancer of activated B cells (NF-κB) transcription factor, particularly when combined with a variety of stimuli, such as senescent cells, obesity, circulating mitochondrial DNA, gut microbiota and diet triggering and sustaining inflammatory conditions (58, 63–68). However, as previously stated, immunosenescence represents the most important contributor to inflamm-aging, in turn, contributing to impaired immune responses. In fact, inflamm-aging is responsible for a high expression of micro (mi)-RNAs that interfere with B cell activation, driving tumor necrosis factor (TNF)-α production and inhibiting B cell activation as measured *in vitro* (69). Increased serum levels of TNF-α are also linked to a defective T cell response, in part due to reduced expression of CD28 (21). Accordingly, in monocytes, the pre-vaccination expression of genes related to inflammation and innate immune response is negatively correlated to vaccination-induced activation of influenza-specific antibody responses (70).

Age-related B cell changes are similar to those observed in T cell compartment and the effects on humoral immune response are detrimental as well. Age also affects B cell numbers and B cell repertoire diversity, as well as immunoglobulin isotypes and receptor repertoire with a decrease in specific humoral immune responses against new extracellular pathogens (71). Activated B cells isolated from older adults display a reduced induction of E47, a class I basic helix-loop-helix protein encoded by the E2A gene. This is the key transcription factor, for the induction of activation-induced cytidine deaminase (AID), involved in class switching and somatic hypermutation. The reduced expression of E2A might be responsible for the decreased avidity of antibodies and diminished antibody-mediated protection (72, 73). This defect might be linked to a reduced interaction with CD40L^+^ T helper cells, because, in older adults, the memory/effector T cells show a reduced expression of CD40L, necessary for B cells cooperation (74). The reduced levels of E47 and AID mRNA in B cells from older individuals are also due to the reduced mRNA stability. It is due to the higher expression of the inflammatory mi-RNAs 16 and 155, which bind to the 3'-untranslated region of E47 and AID mRNA, respectively, inducing mRNA degradation (69). In addition to the decrease in circulating B lymphocytes, there is a shift from immunoglobulin produced by naïve cells (IgD, IgM) to immunoglobulin produced by memory B cells (IgG, IgA). This is accompanied by an impaired ability to produce high affinity protective antibodies against infectious agents and the shrinkage of the repertoire diversity. The reduced serum levels of IgM and IgD suggest a shift in the balance from the naïve (CD27) toward the memory compartment (CD27^+^), although this is not observed in all studies (71, 75–77).

**Figure 1:**
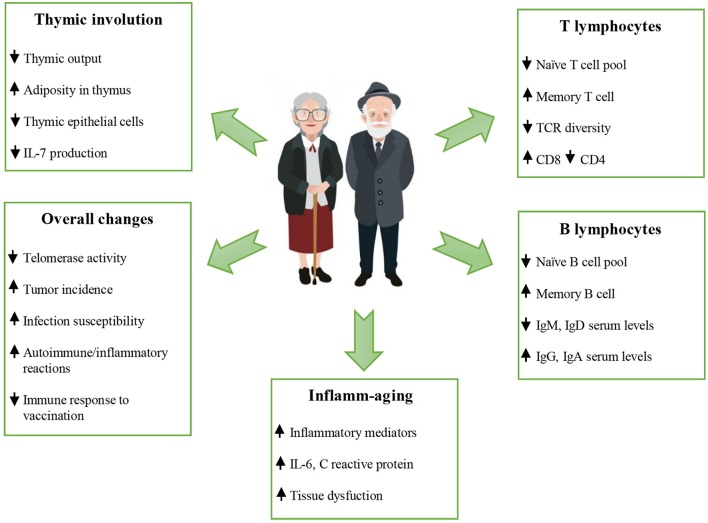
Schematic changes occur during aging. The immunosenescence in older people is characterized by thymic involution, altered T and B cell responses, altered naïve/memory *ratio*, increased serum levels of IgG and IgA, a chronic low grade inflammation, and a poor response to newly encountered microbial antigens, including vaccines. The reduced ability to respond to new antigens, the accumulation of memory T cells and the lingering level of low-grade inflammation “inflamm-aging”) are considered hallmarks of immunosenescence. See the text for the acronyms. 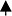 increase; 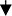 decrease.

See Figure 1 for the schematic changes occurring during aging.

## Dietary Strategies Currently Being Investigated

There is a mutual interaction between nutrition, intake of particular bioactive dietary components, immune function, and inflammatory status, hence a close relation with the hallmarks of immunosenescence (78, 79). Many existing data demonstrate the key role of foods as regulators of immune response and inflammatory status, hence as possible modulators of the rate of immunosenescence, particularly inflamm-aging (11, 80). Other data have demonstrated the importance of following a specific, even personally tailored, dietary pattern (81). However, the intricate cellular and molecular network of immune system makes difficult to identify targeted strategies to rejuvenate specific compartment of immunity. Starting from supplementation with a single nutrient, leading to the application of experimental dietary pattern, much progress has been made in this field. The main barrier to better clarity remains the wide heterogeneity among human beings, linked to different life-style and genetic factors that influence the rate of immunosenescence (6, 10, 11, 82). Data discussed below show that the main target of dietary strategies is inflamm-aging, because diet, biotics, and nutraceuticals can show anti-inflammatory and anti-oxidant properties.

### Diet

The high rate of long living people and the low incidence of cardiovascular disease in many Mediterranean countries suggest the importance of a diet rich in fruits, vegetables, whole grains, legumes, and olive oil (probably the main anti-aging food in this area). The reduced consumption of animal proteins, in particular red and cured meat, is also important. The efficacy of this diet results as an attenuation of inflammation and oxidative stress, and from the maintenance of a condition of eubiosis of the microbiota, involved in the general improvement of immune response in these populations (68, 83–86). In particular, the Mediterranean diet down-regulates the levels of inflammatory mediators, such as soluble intercellular adhesion molecule (ICAM)-1, vascular cell adhesion molecule (VCAM-1), C reactive protein, and IL-6, as well as many other biomarkers of inflammation (87–90).

A new interesting approach related to the possible reversion of immunosenescence is caloric restriction. NF-κB, mechanistic target of rapamycin (mTOR), and MAPK, pathways closely related to aging and inflammation are modulated by caloric restriction that downregulates the activation of IL-1β, IL-6, and tumor necrosis factor (TNF)-α genes, hence the pro-inflammatory state (20, 91, 92). More specifically, the results obtained by administration of different cycles of fasting, mimicking diet or long-term fasting influences inflamm-aging (20). These dietary patterns explicate their activity, particularly, during the refeeding period, reducing the rate of aging because of their antioxidant and anti-inflammatory effects, and possibly counteracting some other aspects of immunosenescence. The hypothetical explanation might be the disposal of damaged cells with growth of new functional cells.

Minor effects on immune function, after a brief starvation period of 72 hours, were seen in ten healthy, normal-weight, young volunteers. They showed an increase in suppressor cell numbers but no change in the number of peripheral blood leucocytes or in the differential counts (93). Unfortunately, these studies are severely limited by their complexity, further confounded by the small number of cases analyzed and the poor participants compliance (81).

New insights may also come from the use of caloric restriction mimetics, such as metformin, an activator of 5′ AMP-activated protein kinase (AMPK). It is a drug, typically administrated in type 2 diabetes but proposed as an anti-aging molecule for humans, such as the study called “Targeting Aging with Metformin” (94). Metformin can trigger AMPK, a pathway activated by energy depletion, i.e., by low levels of intracellular adenosine triphosphate (ATP), leading to the extension of healthy lifespan in model organisms (95). In mice with collagen-induced arthritis, metformin administration had an anti-inflammatory effect on arthritis due to the inhibition of Th17 cell differentiation, a subset of pro-inflammatory cells producing IL-17, and the upregulation of T regulatory cells (Tregs) differentiation along with the suppression of osteoclast differentiation (96, 97). Contrarily to caloric restriction, undernutrition, which is common in older people, is associated with an immunocompromised state, linked to altered T cell numbers, a reduced response to antigens, impaired release ofmediators, such as cytokines, and decreased phagocytosis and NK cell activity. This makes older people enable to trigger an efficient immune response to newly encountered pathogens. In such conditions of poor nutrition, the use of supplements, such as zinc, copper, iron, vitamins, nutraceuticals, and probiotics could be desirable and more appropriate than caloric restriction, as demonstrated by previous studies (98, 99).

### Micronutrients

Nutritional status is crucial for the health status of older adults. Changes in phenotypic features, mainly loss of teeth and alterations in taste receptors, and gut disorders as well, determine a variation in both quality and quantity of food intake, contributing to general alterations in metabolism (100). Many studies have examined the influence of micronutrients and their influence on the enhancement of immune function in older adults (11, 79, 101, 102). Micronutrients, such as vitamins and minerals, are essential for the efficient performance of the immune system. They are needed in trace quantities, because the homeodynamic range is small, but the maintenance of a correct amount and balance is very rare in older people (often even in adults and young), both for scarcity and for excess due to unnecessary supplementation (78, 103).

One of the main micronutrients related to physiologic processes associated with immune system, and one of the main studied factors, is zinc. It is involved in many molecular processes, such as signal transduction, apoptosis, proliferation, and differentiation of cellular components of the immune system. Even slight deficiencies in zinc can have important consequences (104–107). Zinc deficiency can cause decreased levels of serum thymulin, a zinc dependent peptide hormone produced by thymic epithelial cells, with an activity that is progressively reduced with age, with a peak in pre-adolescence (78, 104, 108). The active form of thymulin induces the expression of markers of T lymphocyte activation, promoting T-mediated functions, acting both on the early and on the late phases of lymphocyte differentiation (109, 110). As shown in a randomized, doubleblind, placebo-controlled trial, after zinc supplementation for 12 months (45 mg elemental Zn gluconate/day), the incidence of infections was significantly lower, plasma zinc was significantly higher, and generation of TNF-α and oxidative stress markers was significantly lower in the zinc-supplemented participants than in the placebo group (both groups composed of 55–87 years old persons). Another doubleblind, randomized, controlled trial performed with zinc supplementation in old people (25 mg as zinc sulfate, once a day for 3 months, mean age of placebo group 80.6 ± 7.8, mean age of supplemented participants 79.5 ± 6.8) demonstrated increased levels of activated T helper and cytotoxic T lymphocytes, with a higher relative percentage of T cells with respect to the total circulating lymphocytes in zinc-supplemented older adults (105, 106, 111). Given the dose-dependent effect of zinc, both as a pro- and anti-oxidant, its presence in the normal range is essential for regulating the levels of reactive oxygen species (112). These studies highlight the importance of the zinc for immune function, but contrasting results exist, possibly reflecting the intrinsic complexities of this type of investigation (11).

Vitamin supplementation studies in older adults have demonstrated a role for vitamin E in the production of IL-2 as well as the activation induced T cell proliferation in naïve but not in memory T cells (78, 113–115). However, this response is variable, depending on genetics and immune functionality (102). Moreover, age-related oxidative stress, hence inflamm-aging, can be counteracted by vitamin C supplementation. In addition, it seems that this vitamin is involved in enhanced antibody generation and in differentiation and maturation of immature T-cells as well as of NK cells. Because vitamin C is water-soluble and humans have low storage capacity, its regular intake is up to 100-fold higher than that for many other vitamins (116, 117).

### Probiotics, Prebiotics, and Symbiotics

The use of probiotics, prebiotics and symbiotics, i.e., the combination of pro and prebiotics, as immunomodulators, which act on microbiota, is very common. However, no strong cause-effect relation often exists between their use and specific end-points. Gut microbiota that plays an active part in healthy status, is compromised in older adults due to malnutrition, use of medications and immunosenescence itself. Therefore, the administration of specific strains of *Lactobacilli* and *Bifidobacteria* as probiotics as well as fructooligosaccharides, galactooligosaccharides, and other prebiotics, or the combination of both might constitute a benefit for immunocompromised people (118–122).

Data from supplementation studies with pro- or prebiotics in older adults show a control of inflammatory status because their use is responsible for a lower production of TNF-α, IL-1β, and IL-6 as well as an increase of anti-inflammatory cytokine IL-10, by PBMCs. In addition, these biotics improve the innate immune responses by the modulation of phagocytosis and cytotoxicity against specific bacteria, such as *Staphylococcus aureus*, increase activity of peripheral blood NK cells, and lower CD25 expression by resting T lymphocytes (123). However, the complexity of randomized controlled trials and lack of specific biomarkers in humans make difficult the reproducibility of the data (124). Moreover, healthy status, including absence of disease and nutritional status, seems to be crucial for their action as demonstrated by null results on immunomodulation after administration of prebiotics in older adults vaccinated with influenza or pneumococcal vaccines (125). Further studies are summarized in a very recent review by Suez et al., although in this case too, it is highlighted the weakness of the existing data (124). Therefore, a strong limitation linked to the study of these potential modulators is the lack of mechanistic studies that could reveal the molecular mechanisms underlying their action. This would allow a targeted and effective use, and would reduce the bias linked to individual variability and the conflicting existing results present in literature. Although meta-analyses and systematic reviews report interesting data, they cannot replace multicenter, randomized controlled clinical trials to address the relevance of the use of probiotics or the composition of the microbiota, both accompanied by molecular explanation of the observed evidence.

### Nutraceuticals

Recently, various bioactive food components associated with health-related effects have been called nutraceuticals. These food compounds, mainly found in plant-based foods and fatty fish, have been implicated in offering physiological health benefits over and above basic nutritional requirements (126–128). Now, there is much interest in optimizing the immune response, and in reducing inflammation in older adults by increasing the intake of certain bioactive food agents (129, 130). Many studies have investigated how immune function and inflammation are directly affected by nutraceuticals. They provide evidence that increasing intake of some of them above the habitual and recommended dose levels can enhance some aspects of immune function, and reduce the level of inflammatory status, increasing cellular resistance to aging (131–133). Below, we examine the immunomodulatory effects of three classes of nutraceuticals, namely carotenoids, polyphenols, and polyunsaturated fatty acids (PUFAs), summarizing the most relevant nutritional studies on the reciprocal interactions between these dietary agents and immunosenescence.

Carotenoids are naturally occurring pigments found in most fruits and vegetables. They primarily exert antioxidant, hence anti-inflammatory, effects, but individual carotenoids may also act through other mechanisms, including immune-enhancing activities (134, 135). Jyonouchi et al. observed that lutein and astaxanthin increased the *ex vivo* antibody response of mouse splenocytes to T-cell antigens (136). Older adults supplemented with carotenoids (30 mg β-carotene, 15 mg lycopene and 9 mg lutein) had a shift to T cells expressing a mature phenotype and, in addition, higher IgA serum levels, and an increase in NK cells (137). Watson et al. report that higher doses of β-carotene (30 and 60 mg/day; instead of 15, 30, and 45) increase T helper cells and NK cells number (138). Although higher doses of carotenoids are not easily achievable in the diet of population, these findings suggest that low doses are insufficient to affect immune responses. Enhanced NK cell cytotoxicity was observed in participants treated with oral β-carotene and, similarly, long-term β-carotene supplementation increased NK cell activity in older adults (139, 140).

Dietary polyphenols are the biggest group of phytochemicals and they are defined as bioactive non-nutrient plant compounds. They are in fruits, vegetables, grains, and other plant foods, the consumption of which has been linked to reduction in risk of major age-related diseases (141, 142). In fact, as discussed below, their main action is the control of inflammation. Consumption of cocoa polyphenols rich in flavonoids (40 g/day) with 500 ml of skimmed milk, by participants at high cardiovascular disease risk (≥55 years), significantly reduced the expression of cell adhesion molecule very late antigen-4, CD40, and CD36 on monocytes. This treatment also lowered circulating levels of the inflammatory markers P-selectin and ICAM-1, compared with monocytes from the control group (only skimmed milk) (143). *In vitro* studies have shown that administration of olive oil polyphenols (caffeic acid and oleuropein glycoside) to human whole blood cultures stimulated with lipopolysaccharides significantly reduced IL-1β levels compared with stimulated control cultures that were not incubated with olive oil polyphenols. Interestingly, responses were inversely correlated to the dose (144). A small scale (*n* = 23) pilot study has shown that daily consumption of 12 green olives, containing oleuropein and hydroxytyrosol, significantly reduced serum IL-6 and malondialdehyde (a lipid peroxidation marker) levels after 30 days of consumption by healthy adults (90). Although several reviews have postulated potential beneficial effects of polyphenols on the immune response of older adults, there have been limited studies on this topic (145). However, the major effects of polyphenols are associated with increased release of IL-2 and IFN-γ, hence enhancing immune response (146). For example, resveratrol, a polyphenol typically found in red wine, grape skins, and berries, induces a significant increase in T helper cells and in the delayed-type hypersensitivity response of aged rats (147).

In addition to carotenoids and polyphenols, several studies have also shown that dietary lipids can modulate the immune response. Fatty acids that have this role include the long-chain PUFAs of the omega-3 (n-3) and omega-6 (n-6) classes. n-6 PUFAs, derived from plants and land animals, have minimal effects on immune response. n-3 PUFAs, eicosapentaenoic acid (EPA) and docosahexaenoic acid (DHA), found mainly in fish and fish products and in some plants (flax seeds), have the most significant impact on immune cells. These have anti-inflammatory properties inhibiting the formation of eicosanoids and synthesis of pro-inflammatory cytokines (IL-1β, TNF-α, IL-6), chemokines (IL-8, monocyte chemoattractant protein 1), and adhesion molecules (ICAM-1, VCAM-1, selectins) (148). However, because dose, timing of administration, and participant age are important modulating factors of the effect of these molecules, contrasting results exist and only few studies focus on their use in older adults (149–154).

## Clinical Approaches Currently Being Investigated

### Growth Factors

The various aspects of IL-7 physiology raise the possibility that reduction of this pleiotropic cytokine level could contribute to the age-related decrease in the absolute number of thymocytes and naïve T cells. Therefore, IL-7 might be used as a therapeutic agent to enhance thymopoiesis in lymphopenic patients or in older individuals, so counteracting the first hallmark of immunosenescence, i.e., the reduction of naïve T cells. In fact, the profound structural remodeling that characterizes the thymic involution also affects thymic epithelial cells with a consequent reduction in the intrathymic production of IL-7 (155, 156).

IL-7 produced by thymic epithelial cells provides survival and proliferative signals to immature double negative CD4^−^CD8^−^ thymocytes and promotes V(D)J recombination of the T cell receptor (TCR) γ-c locus (157). Mutations in the IL-7Rα or γc in humans lead to severe combined immunodeficiency, confirming the importance of the IL-7 signaling pathway in the development of T cells (158, 159). At later stages, the IL-7/receptor signaling complex is required for the homeostatic proliferation of naïve T cells in the periphery, exerting a higher effect in the cytotoxic T cell subsets. The high expression of IL-7Rα on naïve T cells allows the maintenance of the pool of these cells, but there are limited amounts of IL-7 under physiological conditions. Following the encounter with its cognate antigen, naïve T cells lose IL-7Rα expression and differentiate into effector T cells. IL-7R down-regulation guarantees an efficient use of the limited amount of IL-7 to naïve T cells that need it, driving their proliferation and preserving their phenotype (160). IL-7Rα is re-expressed at the memory stage, ensuring cell survival and proliferation in memory T cell pool too (156).

Interestingly, IL-7Rα chain is an integral component of the receptor for thymic stromal lymphopoietin (TSLP). TSLP provides normally a co-mitogenic activity that is less potent than that of IL-7 (161). However, to best of our knowledge no study has been performed on the possible role of TSLP in the treatment of immunosenescence.

In the first clinical trial in humans, patients with metastatic cancer (age range 20–59 years) treated with different doses of IL-7 showed a dose-dependent increase in circulating CD4^+^ and CD8^+^ lymphocytes along with a decrease in Tregs (162). Since then, numerous other clinical trials have used the administration of IL 7 for treating patients with various malignancies and chronic viral infections. In HIV-infected patients, with persistently low CD4^+^ T-cell counts despite viral suppression, repeated cycles of recombinant human IL-7 induced a dose-dependent increase in circulating levels of both naïve and memory CD4^+^ and mostly naïve CD8^+^ T cells (163).

Therefore, some data suggest that IL-7 could have a therapeutic potential in improving the clinical outcome in settings that require enhanced immunological responses. However, in the complex scenario of aging, the immunorestorative properties of IL-7 may not be as great as initially hoped, most probably due to the deterioration of the thymus structure. The integrity of cortical and medullary thymic architecture and the presence of functional thymic epithelial cells are required to support and maintain thymopoiesis (164). Therefore, IL-7 effect on T cell development probably should require the preceding restoration of the thymic architecture.

### Checkpoint Inhibitors; The Example of PD-1 and CTLA-4

The role of the immune checkpoint inhibitors, MoAbs that inhibit the expression of certain proteins made by T cells and some cancer cells, or antibodies that block the activation of inhibitory receptors, are pivotal for the management of cancer that occurs both in young and old patients. In fact, immune checkpoint inhibitors promote the immunological control of cancer cells by blocking the immune inhibitory responses that are evolutionary designed to prevent continuing immunological responses once an antigenic stimulus has been eradicated (165). However, there is a gap in the knowledge of the role of immune checkpoint inhibitors in the control of immune response in older patients because the data from randomized clinical trials are conflicting and often lack adequate statistical power.

The PD-1 and the cytotoxic T-lymphocyte antigen (CTLA)-4 are examples of checkpoint inhibitory receptors. The first regulates the inhibition and the fine-tuning of T cell responses. The second is a protein that contributes to the suppressor function of Tregs, mediating the inhibitory effect through the coordinated actions with the co-stimulatory receptor CD28. Activation of CD28 induces on lymphocytes and monocytes the expression of PD-1, which in turn interacts with its ligand (PD-L1) to regulate the balance between stimulatory and inhibitory signals needed for effective immune responses against antigens. This engagement leads to the inhibition of CD28^−^ mediated co-stimulation, hence of TCR-mediated lymphocyte proliferation and cytokine secretion. The modulation of these pathways boosts anti-cancer immunity. Interestingly, the expression of PD-1 increases on T cells of older adults and its blockade partially restores T cells to functional competence (166–168).

The studies we discuss below are clinical studies based on the response to cancer. However, positive clinical data mean an increase in effector cell immune response, i.e., that therapy is in some ways targeting immunosenescence, or at least, dealing with the consequences of immunosenescence. In fact, immunosenescence influences the efficacy of the immune checkpoint inhibitors in older people (169); accordingly, the therapy is less efficient in patients ≥75 years (see below), probably due to a greater degree of immunosenescence. Consequently, there is limited evidence of successful therapy with immune checkpoint inhibitors in older adults, although a few observations of effectiveness in some patients are very encouraging. In the metastatic melanoma, for example, the use of the MoAb Nivolumab, a PD-1 inhibitor, alone or in combination with other antagonists, has survival benefits independently on age (170, 171). In another study, the administration of PD-L1 antibody Atezolizumab also shows positive results for all participants enrolled (172). In these studies, T cells of older adults were still able to respond to the blockade of their inhibitory receptors with a recovery of cytotoxic activity. Moreover, there is evidence about the efficacy of anti PD-1/PD-L1 MoAbs in older patients with non-small cell lung cancer (NSCLC) compared with chemotherapy. The benefit of immunotherapy in terms of response is stackable between younger and older patients (173).

Regarding the CTLA-4 use, several preclinical and clinical trials have reported the role of CTLA-4 inhibition in some kinds of cancer. In particular, the blockade with Ipilimumab can establish an anti-leukemic effect after allogeneic hematopoietic stem cell transplantation and can restore anti-tumor reactivity for patients with relapse (174). Although durable responses were observed, the efficacy of CTLA-4 inhibition needs to be confirmed. However, a recent meta-analysis analyzed the contextual administration of anti-CTLA-4 (tremelimumab and ipilimumab) and anti-PD-1 (nivolumab and pembrolizumab) molecules in four different settings: melanoma, prostate cancer, renal cell carcinoma, and NSCLC. The authors demonstrated a 37% reduction of the risk of death in favor of immune checkpoint inhibitors compared with control arm (175).

Recently, it has been demonstrated that the efficacy of the treatment with immune checkpoint inhibitors can be influenced by the composition of the host gut microbiota (176). As discussed above, the gut microbiota influences the immune system of the host. In fact, the interaction between specific microorganisms molecular pathways and immune cells can regulate local or systemic inflammation, hence influencing immune response (177). In particular, in cancer patients, the gut microbiota dysbiosis, caused by broad-spectrum antibiotic use, can be a contributor to immune checkpoint inhibitors resistance. In one study of 249 patients with NSCLC, renal cell carcinoma, and urothelial carcinoma treated with MoAbs against PD-1/PD-L1, a shotgun sequencing identified an overrepresentation of bacterial genera including *Akkermansia muciniphila* in responders to PD-1 inhibition compared with non-responders. In these patients, lymphocyte reactivity against *A*. *muciniphila* and IFN-γ production was significantly associated with survival (178). The analysis of 112 buccal and fecal samples from patients with metastatic melanoma also showed that the response to anti-PD-1 therapy depends on differences in the diversity and composition of the patient gut microbiota of responders vs. non-responders (179). These data demonstrated that, in responding patients, there was a relative abundance of bacteria of the *Ruminococcaceae* family. Moreover, in mice and patients, T cell responses specific for *Bacteroides* species, such as *thetaiotaomicron* or *fragilis* were associated with the efficacy of CTLA-4 blockade. On the contrary, tumors in antibiotic-treated or germ-free mice did not respond to CTLA blockade (180). Moreover, fecal microbiota composition of 26 patients with metastatic melanoma, using 16S rRNA, at time 0 and before each Ipilimumab treatment, was clustered on microbiota patterns. Baseline gut microbiota enriched with *Faecalibacterium* and other *Firmicutes* was associated with beneficial clinical response to Ipilimumab (181).

With the advent of immune checkpoint inhibitors immunomodulation is going to revolutionize the clinical management of at least some forms of cancer in older patients. In spite of several controversial points, some clinical trials suggest a significant benefit of immunotherapy in older patients, with the exception of patients ≥75 years that obtain less benefit from these treatments. Concerning this point, Metcalf et al. (25) have demonstrated that CD28^−^ costimulation is required for the expansion of PD-1^+^ CD8 T cells and effectiveness of PD-1 therapy in murine models of chronic viral infection and cancer. In addition, in lung cancer patients, PD-1+ CD8 T cells that proliferate in the peripheral blood after PD-1 blockade express CD28. Therefore, these data, which imply selective proliferation of CD28^+^ cells by PD-1 therapy, highlight one mechanistic explanation why cancer patients older than 75 years may not respond as well to immunotherapy as younger patients. Understanding immune-regulatory functions is critical to implement integrative immunomodulatory strategies targeting checkpoints inhibitors.

Further studies of these checkpoints inhibitor functions might provide to be of great therapeutic value also in improving T cell responses to boost anti-microbial immunity and vaccine efficacy during aging as well. The combination of immunological, biochemical and systems biology data provides significant support for using PD-1 as an important target for therapeutic interventions of this type. In fact, studies carried out on HIV, hepatitis B and hepatitis C infections have shown that blocking the interaction PD-1/PD-L1 has a positive effect on the effector functions of T cells. Furthermore, future studies focusing on the elucidation of additive effects of blocking PD-1, other negative regulatory molecules, and immunosuppressive cytokines will help to identify combinatorial approaches that can improve T effector responses to vaccination and therapeutic interventions in older patients (182).

### MAPK Pathway; Focus on p38 Regulation

Recently, the role of MAPKs pathways in the functional competence of the immune system has been demonstrated (183). The MAPK signaling pathways have been extensively studied in the context of oncogenic function and proliferative stimulus. However, these complex systems also regulate several functions of the innate and acquired immunity. They are also involved in the production of pro-inflammatory cytokines, as well as in the intracellular signaling cascades initiated when a cytokine binds to its corresponding receptor (183). Three main subgroups of MAPKs are known: Erk, Jnk, and p38. These kinases can be targeted by small molecular weight compounds, which act to inhibit the phosphorylation of proteins, hence preventing their activation. Each one is separately regulated within individual cells (184) [for an overview of kinase inhibitors see (183)]. Understanding the immune-regulatory functions exerted by MAPK pathways is critical to implement integrative immunomodulatory strategies targeting these kinases.

The p38-MAPK pathway plays a pleiotropic role in cell survival, both sustaining proliferation, and inducing apoptosis in a cell type-specific manner, depending on the type of stimulus (185). The p38-MAPK pathway stimulates the positive regulation of Th1 differentiation and polarization. This pathway is not active in Th2 cells (186). The p38-MAPK pathway is critical for the production of inflammatory cytokines, positively regulating the production of IFN-γ in CD4 and CD8 cells (187, 188).

The studies discussed below have been performed *ex vivo* in mononuclear cells from mice and humans. They point out the possibility to affect the second hallmark of immunosenescence (the accumulation of memory T cells) through the regulation of p38 activation. p38 is generally absent in senescent human T cells. However, IFN-α signal can activate p38, triggering cellular senescence, and leading to inhibition of proliferation and telomerase activity in non-senescent T cells (189). It is also associated with alterations of energetic metabolism as well as autophagy. Autophagy, by inhibiting cell senescence, is a critical regulator of memory CD8^+^ formation, and age-related autophagy defect is one of the explanations why CD8^+^ T memory formation becomes defective in old age (38, 185). In 2009, Eisenberg et al. identified the use of spermidine, a polyamine compound, to promote longevity, via autophagy, using PBMCs as model. The authors monitored the survival cells using annexin V/7-AAD as co-staining. After 12 days, 50% of the cells survived after addition of spermidine. The rescuing effect did not involve inhibition of apoptosis, as the percentage of apoptotic cells was not influenced by spermidine. In fact, cell death, associated with membrane rupture, was indicative of necrosis (190). In immunosenescence models, CD8^+^ T cell can be also rejuvenated in an autophagy dependent manner, using spermidine (191, 192). Low doses of a synthetic compound of natural spermidine significantly suppressed autophagy in human Jurkat T cell line. Moreover, the use of spermidine dramatically improved the CD8^+^ T cell response to vaccination and infection in aged mice in an autophagy-dependent manner, contributing to the increased numbers of antigen-specific CD8^+^ T cells (191).

Moreover, the effector memory CD8^+^ T cells that express CD45RA, are not functionally exhausted. Indeed, they preserve the ability to secrete high levels of specific cytokines, such as IFN-γ and TNF-α. Furthermore, they only express low levels of key markers of exhaustion, such as PD-1. In these cells that present characteristics of immune senescence (decreased proliferation, lower telomerase activity, and increased presence of DNA damage), the simultaneous blockade of both p38-MAPK and PD-1 signaling supports their proliferation, both in young and in older human beings. Secretion of TNF-α in some populations of cells is reduced because of the contemporary arrest of p38-MAPK and PD-1 pathways. However, the telomerase activity in CD8^+^/CD45RA^+^ T cells is improved by blocking only the p38 pathway but not the PD-1 signaling, indicating that non-overlapping signaling pathways are involved (193, 194).

In addition to the inflammatory pathways that activate p38 through MAPK cascade by auto-phosphorylation, p38 can be associated with AMPK complex in response to chronic antigenic stimulation (see below, next paragraph).

The success of the studies using MAPK inhibitors, and kinase inhibitors in general, allows the possibility to analyze, and discover, the potential of these molecules in the treatment of immunosenescence, targeting the second hallmark. For example, a block at the level of p38-MAPK by sestrins causes age-related signaling defects in effector and memory CD45RA^+^/CCR7^−^ T cells (195, 196). Sestrins, the mammalian products of the Sesn1, Sesn2, and Sesn3 genes, are a family of stress sensing proteins (196). Lanna et al. proposed a possible role for sestrins in the control of the immune response, although this role has not yet been fully determined. Sestrins exhibit pro-aging activities in T senescent lymphocytes. The authors identified a complex named sestrin-dependent MAPK activation complex (sMAC) that simultaneously coordinates the activation of each MAPK that controls a functional response. The knockout of sMAC restored T cell activity (antigen-specific proliferation and cytokine production) from older humans, and enhanced responsiveness to influenza vaccination in the aged mice (196).

### Examples of Nutrient Signaling Pathways: AMPK and mTOR

The mechanisms exposed above are distinct from another sestrin-inhibitory complex, containing GATOR and RAG A/B GTPase that involves the mTOR pathway (197–199). In particular, sestrins stimulate the activation of AMPK (by an unknown mechanism), inhibiting mTORC1 signaling. This suggests that the anti/pro-aging dichotomy of sestrin action in T cells vs. other cell types may depend on different sestrin-protein interactions (200).

In turn, senescent human CD27^−^/CD28^−^/CD4^+^ T cells trigger AMPK to stimulate p38 recruitment, causing p38 auto-phosphorylation mediated by the protein scaffold TAB1. This pathway can inhibit telomerase activity, T cell proliferation, and expression of key components of the TCR signalosome. In the presence of low-nutrient levels and DNA-damage signaling the proliferative defect of senescent T cells is reversed by blocking AMPK-TAB1-dependent p38 activation (38). Moreover, in senescent CD8^+^ T cells, p38-MAPK induces an increase in autophagy through interactions between a p38 interacting protein and autophagy protein 9, in a mTOR-independent manner, suggesting that p38-MAPK blockade reverses senescence via mTOR-independent pathway (185).

mTOR plays an important role in T cell activation and differentiation, especially of naïve CD4^+^ T cells in their differentiation toward Th1 or Th17 phenotypes (201, 202). The activation of mTOR signaling pathway is under the control of TCR/CD28 stimulation (201, 203). A growing body of research has highlighted mTOR inhibitors, i.e., rapamycin and everolimus, as promising treatments for several age-related pathologies, including immunosenescence, prolonging lifespan, especially in all four major animal models of aging: yeast, worms, flies, and mice (204, 205). The partial inhibition of mTOR could be beneficial for immune function in older people, although mTOR activity inhibits autophagy. At high doses, rapamycin is immunosuppressive, blocking both protein synthesis and cell division. In a clinical trial of over 200 older participants, they were assigned to a protocol including the use of mTOR complex 1 inhibitor everolimus, in different daily doses, for a 6-weeks period. Participants, after a 2-weeks drug-free interval, were challenged with the seasonal influenza vaccine. The two low-dose everolimus regimens improved immune function without causing serious side effects. Patients ameliorated their immune response, with improved hematopoietic stem cell function and a decreased proportion of PD-1^+^ lymphocytes (206). In a subsequent follow-up study, combined BEZ235 (a dual ATP-competitive PI3K and mTOR inhibitor) and everolimus treatment for 6 weeks resulted in better infection control in older adults for a year after treatment had ended (207). However, rapamycin and Torin, another mTOR inhibitor, are also reported to suppress the anti-inflammatory effects of circulating glucocorticoids (208). These findings conflict with earlier studies showing the central importance of mTOR in innate immunity, specifically in the production of anti-inflammatory IL-10 and the suppression of pro-inflammatory cytokines IL-21 and IL-1β (209). The improved response after rapamycin treatment, which might involve a decrease in the percentage of PD-1 positive T cells, requires more detailed studies (207).

Data suggesting that nutrient signaling pathways may negatively influence lymphocyte function in aging indicate the possibility that inhibition of these pathways may enhance the activity of lymphocytes from older adults (210). Broad ranges of pharmacological agents with anti-immunosenescence properties have been identified and other trials with agents, such as rapamycin analogs are underway. Therefore, this represents a promising therapeutic approach to improving the health of older adults.

**Figure 2:**
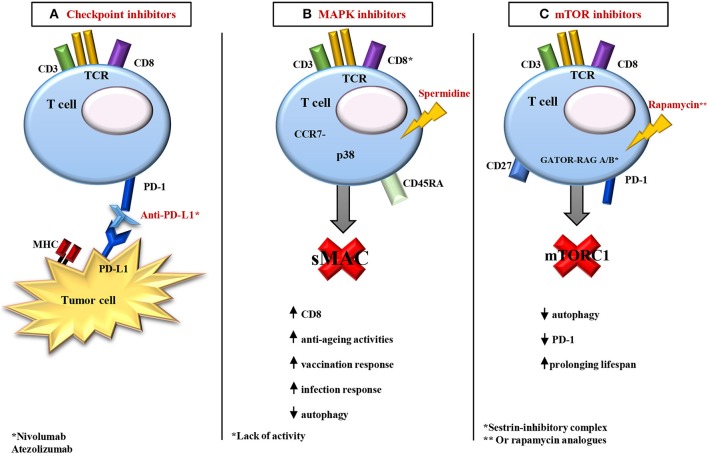
Overview of the main strategies of immunomodulation. **(A)** In age-related diseases, in particular in some metastatic cancers, the use of the monoclonal antibody (such as Nivolumab, a PD-1 inhibitor) alone or in combination with other antagonists (PD-L1 antibody, like Atezolizumab) shows positive results on T cell activities in older adults. These cells became able to respond to the action of their inhibitory receptors with a recovery of cytotoxic activity. **(B)** In old models, with Lack of activity of CD8^+^, it is possible to rejuvenate CD8^+^ T cell responses in an autophagy dependent manner, using the polyamine compound spermidine. Spermidine induces autophagy and prolongs lifespan in model organisms. Moreover, in the CD8^+^/CD45RA^+^/CCR7^−^ T cells, a block at the level of MAPK p38 by sestrins causes age-related signaling defects. The knockout of sMAC restores T cell activity (antigen-specific proliferation and cytokine production) in older humans, and enhances responsiveness to influenza vaccination in old mice. **(C)** GATOR and RAG A/B GTPase make a sestrin-inhibitory complex that involves the mTOR pathway. In particular, sestrins stimulate the activation of AMPK (by an unknown mechanism), inhibiting mTORC1 signaling. In addition, mTOR inhibitors, i.e., rapamycin and everolimus, are promising treatments for several age-related pathologies, including immunosenescence, prolonging lifespan. A soft inhibition of mTOR could be beneficial for immune function in older adults, although mTOR activity inhibits autophagy, and involves a decrease in the percentage of PD-1 positive T cells. See the text for the acronyms.

See Figure 2 for the main clinical approaches in immunomodulatory interventions.

## Other Approaches in Development

Other approaches focus on development of novel vaccines especially suited to raise protective immunity in older adults by overcoming the decrease in naïve cells. This approach includes high-dose vaccines, booster vaccinations, different immunization routes, and use of new adjuvant. The most used adjuvants are based on aluminum salts. These adjuvants induce the activation of APCs and strengthen the antigen immunogenicity by their slower release and higher persistence at the vaccination site. Another interesting compound is MF59, a squalene-based adjuvant, which increases the chemokine-dependent recruitment of APCs (211). However, adjuvants have shown only modest success (212). The most effective is generally considered complete Freund adjuvant, which can only be used in animals because it can cause a damaging skin inflammation (213). Therefore, there is an unmet need for new vaccine strategies for older people.

The development an identification of appropriate adjuvants and cytokines might effectively remedy defects in T cell functions from older adults, both directly and by better activation of DCs (214–216). Stimulation of TLRs by agonists seems to be a promising strategy to enhance vaccine efficacy, because TLR triggering can induce the production of cytokines by APCs, and can promote germinal center antibody production (217, 218). Age-related variations in cytokine production are seen in the APC isolated from older donors and efficient TLR stimulation may overcome the age associated TLR signaling dysfunction (219).

Triggering of multiple TLRs, using a combined adjuvant for synergistic activation of cellular immunity (CASAC), is an intriguing strategy. CASAC incorporates CpG (a single-strand oligodeoxynucleotide, characterized by motifs containing cytosines and guanines), which is a potent inducer of IFN-α by pDCs, in combination with polyI:C (a synthetic analog of viral dsRNA that targets TLR3, inducing the production of type I IFNs). CASAC also contains IFN-γ and MHC-class I and II peptides. This formulation results in potent cytotoxic T cell-mediated immunity in young mice. In fact, immunization with two or more TLR agonists, an activator anti-CD40 antibody, IFN-γ, and surfactants were sufficient to drive unprecedented levels of CD8 responses to peptides or protein antigens and highly polarized Th1 CD4 responses. CASAC stimulation activates both mDCs and pDCs with IL-12 secretion. This strategy is more effective than existing adjuvants and provides a technological platform for rapid vaccine development (213).

Accordingly, in aged mice, antigen specific CD8^+^ T cell responses were stimulated after serial vaccinations with CASAC and a class I epitope, deriving either from ovalbumin or the melanoma-associated self-antigen, tyrosinase-related protein-2. Pentamer analysis revealed that aged, CASAC-vaccinated, animals had substantially higher levels of antigen specific CD8^+^ T cells compared with mice vaccinated with complete/incomplete Freund adjuvant. Therefore, CASAC promoted significantly better functional CD8^+^ T cell activity (220).

An approach able to overcome age-related defects in CD4 T cell responses *in vivo* comes from the ability of combined TLR ligands to induce the activation of peripheral blood DCs isolated from older healthy donors (29). Preliminary *in vitro* screening experiments suggest that, from the various TLR agonists tested, the condition that most effectively activates DCs is the combination of TLR7/TLR8 with TLR4. This TLR agonist combination induces significantly greater cytokine production than that induced by each of the individual agonists. This greater stimulation is probably due to the combined activation of both MyD88 and TRIF-dependent signal transduction pathways. Stimulation with the specific combination of TLR agonists, the imidazoquinoline R848 that targets TLR-7 and the monophosphoryl lipid A that targets TLR-4, induces significantly higher cytokine secretion by mDCs and pDCs from older adults. This has potentially important implications, because the reduced production of cytokines by pDCs from older people, caused by defects in TLR signaling pathways, is associated with an ineffective antibody response to influenza vaccination (221). These findings highlight the efficient effect of adjuvants in the stimulation of cytokine production and point toward the potential use of appropriately selected combination of TLR agonists in future vaccination approaches for older adults to overcome the CD4 inability to respond.

## Conclusion and Future Approaches

Until a few decades ago, a very small fraction of the population would reach 80 years of age. Now, in the Western world, this is a frequent event, with the average life expectancy for a newborn to have risen to 80 years in most Western European countries (1). However, the increase in lifespan does not coincide with increase in healthspan. The link between aging and disease is in part a reflection of the functional changes in the immune system of older people. Different factors contribute to the development of age-related immune dysfunction, but the epilog of an aged immune system is an increased propensity toward a reduced resistance to infection, poorer responses to vaccination, and the development of age-related diseases. The analysis of the contributing factors to this profound immune remodeling has revealed a complex network of alterations that influence both innate and acquired arms of the immune system. The diversity of cells, molecules and pathways involved in this remodeling, and their ability to influence each other, including the intra- and inter-individual variability of the immune response, make it hard to identify interventions that can be predicted to improve or, at least, to maintain the immune function in older adults. Within the past few years, numerous studies of the underlying mechanisms of age-related immune decline have laid the groundwork for the identification of targeted approaches; some of these have been discussed above, focusing on interventions able to target the hallmarks of immunosenescence. Possible other future approaches are reported below.

Taking into account the role of HCMV in the decrease of naïve T cells and increase of memory T cells, the reduction of the latent/lytic viral load, by vaccination and/or antiviral drugs, should be beneficial to diminish HCMV-associated immunosenescence. Concerning the HCMV vaccine, Plotkin has published an extensive review. As pointed out by the author, as a result of 40 years of work, there are many candidate HCMV vaccines, including live recombinants, replication-defective virus, DNA plasmids, soluble pentameric proteins, peptides, virus-like particles and vectored envelope proteins. Therefore, we know the antigens needed in a HCMV vaccine, and that vaccination can be protective. To reach the goal of an effective HCMV vaccine, now we need a concentrated effort to combine the important antigens and to generate durable responses that will protect for a significant period. Interestingly, Plotkin emphasizes that aside from the two main targets for disease prevention, i.e., congenital infection and post-transplant disease, immunosenescence might be a target for vaccination mediated intervention, as well (222). Letermovir is an antiviral agent that inhibits HCMV replication by binding to components of the terminase complex. In patients undergoing hematopoietic stem cell transplantation, Letermovir daily prophylaxis is effective in preventing clinically significant HCMV infection when used through day 100 after transplantation, with only mild toxic effects and with lower all-cause mortality than placebo (223). However, there is no suggestion yet for the use of antiviral therapy as a strategy for prophylactic mitigation of immunosenescence.

Finally, possible future strategies to combat immunosenescence are represented by cellular and genetic therapies, including bone marrow transplantation and genetic reprogramming. In particular, genetically reprogramming cells into induced pluripotent stem cells can rejuvenate any cell type through telomere elongation, overcoming hurdles of replicative senescence (224).

## Summary

In the first part of the review we define immunosenescence and its relevance for the health of older persons, particularly in the context of acquired immunity. In the second part of the review we focus on the possible treatments to mitigate immunosenescence. First, we pay great attention to positive and negative effects of nutrition on immunosenescence. Then, we analyze the possible immunotherapeutic role of interleukin-7 as well as of checkpoint and mitogen-activated protein kinases inhibitors. Finally, we discuss a possible immunotherapeutic intervention to enhance the response of older adults to vaccines, i.e., the use of toll like receptor agonists. Therefore, we present a comprehensive review of several possible therapeutic interventions to alleviate immunosenescence.

## Author Contributions

AA, CC, SD, ML, and GA contributed to draft the manuscript. AA, FF, GC, CC, SD, CG, ML, NZ, and GA contributed to revising it. AA, CC, and GA wrote the final version.

## Conflict of Interest

The authors declare that the research was conducted in the absence of any commercial or financial relationships that could be construed as a potential conflict of interest.
